# Unchanged Erythrocyte Profile After Exposure to Cryogenic Temperatures in Elder Marathon Runners

**DOI:** 10.3389/fphys.2018.00659

**Published:** 2018-05-30

**Authors:** Jadwiga Szymura, Magdalena Wiecek, Marcin Maciejczyk, Joanna Gradek, Malgorzata Kantorowicz, Zbigniew Szygula

**Affiliations:** ^1^Department of Clinical Rehabilitation, Faculty of Motor Rehabilitation, University of Physical Education, Krakow, Poland; ^2^Department of Physiology and Biochemistry, Faculty of Physical Education and Sport, University of Physical Education, Krakow, Poland; ^3^Department of Theory and Methodology of Athletics, Faculty of Physical Education and Sport, University of Physical Education, Krakow, Poland; ^4^Faculty of Physical Education and Sport, University of Physical Education, Krakow, Poland; ^5^Department of Sports Medicine and Human Nutrition, Faculty of Physical Education and Sport, University of Physical Education, Krakow, Poland

**Keywords:** whole-body cryotherapy, elder men, physical activity, hemolysis, erythropoietin, interleukin-3

## Abstract

**Objective:** Endurance runners may experience “sports anemia” resulting from intravascular hemolysis. In addition, aging has negative impact on hematopoiesis and rheological properties of blood, and erythrocyte membranes in older people are more vulnerable to oxidative damage, which together can lead to anemia. Whole-body cryostimulation (WBCST) is increasingly used in the elderly as a method of biological regeneration of athletes or therapy and preventive treatment. That is why the aim of the study was to determine whether repeated WBCST had an effect on the erythrocyte system in master marathon runners, compared to non-training men.

**Methods:** Ten marathon runners (men aged 55.9 ± 5.5 years, training experience 6.71 ± 5.79 years) and 10 non-training (men aged 62.0 ± 5.8 years) were subjected to a series of 24 WBCST (3 min, -130°C) performed every other day. Erythrocyte levels, interleukin-3 (IL-3), erythropoietin (EPO), haptoglobin, bilirubin, and extracellular hemoglobin (HGB_ecf_) concentrations were determined in the blood before and after 12, 24 WBCST, as well as 7 days after their completion.

**Results:** The concentrations of EPO and IL-3 were significantly increased 7 days after the completion of WBCST in both groups (*P* < 0.05). The erythrocyte content and indicators, the bilirubin, haptoglobin, and HGB_ecf_ levels in each group did not change as a result of WBCST. In order to document hemolytic changes and/or factors affecting the severity of erythropoiesis, correlations between growth erythropoietic factors, erythrocyte and hemolytic factors as well as mutual correlations between hemolytic indexes were calculated. There was a positive correlation (*P* < 0.05) between the EPO and IL-3, bilirubin, mean corpuscular hemoglobin, and red blood cell distribution width – standard deviation. There was also a positive correlation between the concentrations of bilirubin and HGB_ecf_, and a negative correlation between haptoglobin and HGB_ecf_ as well as bilirubin concentrations.

**Conclusion:** WBCST treatments, repeated every other day, do not cause hemolytic changes in elder men with high or low physical activity. But also, they are a procedure that does not increase the level of erythrocytes or their hemoglobinization. In athletes, it is not a form of doping. The positive correlation between EPO and bilirubin may be indicative of, for example, the mutual antioxidative effect of these factors.

## Introduction

Currently, whole-body cryostimulation (WBCST) is a method being more and more frequently used in sports medicine and in post-exercise recovery in people practicing various sports disciplines (Banfi et al., 2010; Bouzigon et al., 2016). In elder individuals, WBCST is also applied as a supplement to treatment. In clinical practice, analgesic, anti-inflammatory, and anti-swelling effects of cryogenic temperatures are used primarily in rheumatology and neurology (rheumatoid arthritis, fibromyalgia, ankylosing spondylitis, chronic lower back pain, multiple sclerosis). Improvement in the mental state of people with depressive and anxiety disorders subjected to a cycle of WBCST treatments was noted (Lubkowska, 2012; Bouzigon et al., 2016).

During WBCST, the body is subjected to short-term (up to 3 min) but usually repeated exposure to air cooled down to cryogenic temperatures (-100 to -160°C) (Lubkowska, 2012; Bouzigon et al., 2016). WBCST induces multidirectional biochemical changes in the body, depending on the number and duration of treatments (Lubkowska, 2012; Lombardi et al., 2017). As a result of WBCST, beneficial metabolic changes were found, including reduction of triglyceride concentration, total cholesterol, and LDL-cholesterol, as well as increased HDL-cholesterol concentration (Lubkowska et al., 2010; Ziemann et al., 2013). Multiple WBCST treatments induce immunological changes. After WBCST, an increase in the level of anti-inflammatory interleukins (ILs), such as IL-6, IL-10, IL-1ra, and a decrease in the level of proinflammatory ILs, such as IL-1α, IL-1β, IL-2, IL-8, TNF-α were detected (Banfi et al., 2009a; Lubkowska et al., 2011; Pournot et al., 2011; Ziemann et al., 2012; Mila-Kierzenkowska et al., 2013). Cryostimulation also increases antioxidative defense (Wozniak et al., 2007a; Lubkowska et al., 2015).

Whole-body cryostimulation is used in athletes to accelerate muscle regeneration as well as to weaken post-exercise inflammatory reactions (Pournot et al., 2011; Ziemann et al., 2013; Ferreira-Junior et al., 2014; Costello et al., 2015; Bouzigon et al., 2016; Lombardi et al., 2017). WBCST causes the interaction of soluble intercellular adhesion molecule-1 (sICAM-1) on leukocytes to be reduced. As a consequence, less neutrophils, monocytes, and lymphocytes migrate to injured muscles. The release of reactive oxygen species and proinflammatory ILs by leukocytes is reduced. This may be a mechanism by which WBCST weakens the inflammatory response as a result of muscle damage (Ferreira-Junior et al., 2014). The concentration of sICAM-1 and, at the same time, the activity of intramuscular enzymes demonstrating the damage of myocytes: creatine kinase (CK) and lactate dehydrogenase, were reduced in athletes after the WBCST series (Banfi et al., 2009a). It was also found that lysosomal enzymes activity and CK activity in the blood were lower when the training was preceded by cryostimulation, which indicates that cryogenic temperatures stabilize the lysosomal membranes and reduce the micro-injuries of muscle fibers caused by exercise (Wozniak et al., 2007b). In addition, oxidative stress induced by training is reduced by earlier exposure to cryogenic temperatures, which reduces the susceptibility to microdamage (Wozniak et al., 2007a).

The condition for achieving good results by athletes training endurance disciplines, such as marathon runners, is high aerobic performance, expressed as maximal oxygen uptake (Basset and Howley, 2000). While one of the important determinants of maximum oxygen uptake is the oxygen capacity of the blood, which is, among others, dependent on hemoglobin (HGB) content (Green et al., 1999). Thus, maintaining the correct number and morphological characteristics as well as the hemoglobinization of erythrocytes is of great importance for athletes, especially in endurance disciplines, where “sports anemia” is most frequently found (Mairbaurl, 2013).

The cause of “sports anemia” is erythrocyte damage due to destruction of cell membranes as a result of compression during passage through capillaries within working muscles, osmotic stress or membrane lipid peroxidation as a result of oxidative stress and post-exercise inflammatory processes (Robinson et al., 2006). Among athletes, endurance runners seem to be the most exposed to intravascular hemolysis in addition due to mechanical damage associated with running called “foot-strike hemolysis” (Shaskey and Green, 2000; Fazal et al., 2017).

The primary response to the use of cryogenic temperatures are vasomotor reactions consisting of the contraction of cutaneous blood vessels during WBCST, and then, their vasodilatation after the procedure (Lubkowska, 2012). Erythrocyte membranes squeezing through the blood vessels, contracted by cryogenic temperatures, may undergo mechanical damage (Charkoudian, 2003). This is especially true in case of elder people. Aging has a negative effect on changes in blood rheological parameters, there is an increase in fibrinogen levels, a decrease in deformability and increase in the ability to aggregate red blood cells (RBCs), which contributes to higher blood viscosity and reduced blood flow rate through blood vessels, thus, providing favorable conditions for damage to cell membranes (Kempinska et al., 2017). The aging process may further affect hematopoiesis by altering the production of hematopoietic ILs and decreasing the production of erythropoietin (EPO) in the kidneys as well as increasing the oxidative damage to erythrocyte membranes, which in turn may lead to anemia of a variety of etiologies (Kedziora-Kornatowska, 2005).

The results of research regarding the influence of WBCST on the erythrocyte system are not unambiguous. Both a decrease in the number of erythrocytes and reticulocytes, HGB, and hematocrit (HCT) values (Lombardi et al., 2013), as well as their elevated levels (Stanek et al., 2006) or no significant changes in their level (Ziemann et al., 2012, 2013; Sutkowy et al., 2014) have been noted after WBCST. Changes in the number of RBCs and HGB concentration as a result of WBCST may result in a change in the oxygen supply to cells, affecting the aerobic performance (Basset and Howley, 2000). A decrease in RBCs content and HGB concentration can intensify “sports anemia” in training individuals. However, an increase in their level may increase the oxygen capacity of the blood (Banfi et al., 2009b, 2010; Mairbaurl, 2013), which along with exceeding the accepted values, could qualify WBCST as a prohibited doping method in sports. In the case of non-training elder individuals, damage to erythrocytic membranes as a result of WBCST may cause hemolytic anemia. In turn, in the case of beneficial effects, WBCST treatments could be supplementary therapy in elder people with decreased HGB and HCT levels.

There is no scientific research answering the question of how WBCST treatments influence the erythrocytic system in elder individuals of varying physical activity (PA) levels. Therefore, the purpose of our study was to evaluate whether repeated WBCST treatments have effects on the erythrocyte system in master marathon runners, compared to elder non-training males. It was evaluated whether WBCST undergone every other day, 12 and 24 in total, induce hemolysis and/or have a positive effect on erythropoiesis by increasing IL-3 and EPO concentrations. To our knowledge, this is the first study involving master runners. We hypothesized that multiple exposure of the whole body to cryogenic temperature, but applied every other day, does not cause significant hemolysis, but at the same time, it stimulates the synthesis of IL-3 and EPO in elder males, regardless of their level of PA.

## Materials and Methods

### Participants

The study participants comprised of 20 men aged 50–70 years, divided into two groups differing in PA. The active group was made up of 10 amateur marathon runners [age 55.90 ± 5.51 years, body mass index (BMI) 24.87 ± 1.28 kg/m^2^]. The non-training group consisted of 10 men leading a sedentary lifestyle (age 62.00 ± 5.75 years, BMI 27.44 ± 2.44 kg/m^2^). Training experience in the marathon group totaled 6.71 ± 5.79 years. The marathon runners declared participation in three to five training sessions per week, each lasting 60–90 min, including approximately 60 min of resistance training. During the running training sessions, the subjects covered the distance of 20–50 km per week. The men did not undergo WBCST for at least the last 6 months. They were non-smokers who did not use dietary supplements and did not take any medication. The participants were asked to maintain their current diet and PA, and not to use any wellness treatments (e.g., hydrotherapy, sauna, massage, cold-water baths, and local cryostimulation). The men did not participate in sports competitions during the study period. The characteristics of the subjects are shown in **Table 1**.

**Table 1:** Somatic characteristics of study participants (mean ± SD).

Variables	Marathon group	Non-training group	*P*-value
Age (years)	55.90 ± 5.51	62.00 ± 5.75	0.03
Body height (cm)	175.20 ± 6.37	168.70 ± 8.17	0.06
Body mass (kg)	76.33 ± 6.37	77.97 ± 7.75	0.61
Lean body mass (kg)	60.11 ± 6.62	57.15 ± 6.64	0.33
Body fat (%)	21.33 ± 4.36	26.67 ± 5.30	0.02
Body mass index (kg/m^2^)	24.87 ± 1.28	27.44 ± 2.44	0.01

*P < 0.05 – significant differences marathon runners vs. non-training men*.

The study was conducted in accordance with the Declaration of Helsinki. The methodology of the study was approved by the Bioethical Committee of the Regional Medical Chamber (127/KBL/OIL/2013). Participants were informed about the purpose and the course of the study, and they expressed their consent to voluntary participation in the study. Participants underwent a medical qualification procedure in order to eliminate medical contraindications for the use of WBCST procedures (Lubkowska, 2012). The medical examination included: (1) blood analytical studies (blood morphology, lipid profile, glucose, and percentage of glycated HGB); (2) electrocardiogram analysis, systolic and diastolic blood pressure measurements; (3) medical history. Viral hepatitis type B or C and HIV were excluded in the study subjects. During blood collection and analysis, standard procedures while maintaining the safety measures envisaged for working with potentially infectious material were followed.

### Somatic Measurements

The height of the test subjects was measured with a Martin (United States) type anthropometer to the nearest 1 mm. Body mass and body composition: lean body mass, fat mass, and percentage of body fat were determined on the basis of bioelectrical impedance analysis (BIA) using the eight-electrode (two electrodes placed in hands and two beneath feet), multi-frequency (5, 50, and 200 kHz) Jawon IOI-353 Body Composition Analyzer (Korea). Measurements were performed in accordance with manufacturer guidelines. The BIA reasonably estimates body composition in controlled conditions for healthy and euvolemic adults. It is a high correlation (0.88) between the results obtained by this method, and the results obtained by DEXA (dual-energy X-ray absorptiometry) (Sun et al., 2005). The measurements were taken in a standing position, at the angle of approximately 30° between the elbows straightened in the upper limbs and the trunk. The subjects were dressed only in underpants. The skin of the hands and feet were degreased and dried before the measurements, the electrodes disinfected each time. BMI was calculated for each participant. The characteristics of the somatic build are presented in **Table 1**.

### Physical Activity

The PA of the subjects was assessed using the Seven-Day Physical Activity Recall questionnaire (Sarkin et al., 1997). The participants were instructed on how to complete the questionnaire. According to the assumptions, before the onset of WBCST treatments, the participants assessed their PA in three intensity categories: moderate (4 MET), hard (6 MET), very hard (10 MET), qualifying the efforts to the following which elicit subjective sensations accompanying: fast-march, slow run, and fast run. Strength exercises were also qualified into the very hard category (Sarkin et al., 1997). The assumption was made that 1 MET is equal to 3.5 mL O_2_/kg/min of consumption, which corresponds to the energy expenditure of 1 kcal/kg/h. The 24 h energy expenditure related to the PA in the group of marathon runners (1041.11 ± 347.90 kcal) was significantly higher (*P* < 0.05) than in the non-training group (376.16 ± 439.21 kcal). The males from the marathon group devoted 4.06 ± 3.16 and 5.33 ± 3.70 h per week (respectively) to very hard and hard PA. Men from the non-training group did not declare performing any exercise of very hard intensity, and the time spent on PA of hard intensity was 0.7 ± 1.25 h per week and was significantly shorter than in the active group (*P* < 0.01). The weekly exercise of moderate intensity was not significantly different in both groups and was 6.17 ± 1.92 and 4.18 ± 3.43 h, respectively in the marathon and non-training group.

### Diet Analysis

During the tests, the participants maintained their usual diets without introducing any intentional modifications, they did not use dietary supplements containing macro- and micronutrients, or vitamin supplements. The diet was evaluated in terms of the daily calorie content and percentage of proteins, fats and carbohydrates in the energy demand using the Diet 5.0 computer program (Institute of Food and Nutrition in Poland), based on the 7-day dietary diaries kept by the respondents, based on the atlas of photographs depicting food products and dishes (Szponar et al., 2012). The daily calorific value of the diet and the percentage of individual components did not differ significantly in the compared groups (*P* > 0.05) and amounted to: in the marathon group 3072.8 ± 382.2 kcal/day, protein 16.8 ± 3.3%, fats 33.7 ± 6.6%, carbohydrates 48.9 ± 7.8%; in the non-active group 2898.0 ± 338.9 kcal/day, protein 15.3 ± 2.7%, fats 37.3 ± 6.4%, carbohydrates 47.8 ± 6.6%.

### Whole-Body Cryostimulation Procedure

Each participant was subjected to 24 WBCST treatments over a period of 8 weeks at a certified medical facility. WBCST was performed in a cryo-chamber consisting of two connected rooms (vestibule and main chamber) with controlled temperature and humidity conditions. WBCST was performed three times a week (Monday, Wednesday, and Friday) in the afternoon (2–4 p.m.), 2 h after a meal. The same four individuals were subjected to single treatments, maintain the same time of the treatment during consecutive days. Blood pressure was measured in all subjects directly before the WBCST procedure, and the accepted level of 150/90 mmHg to conduct the treatment was not exceeded by any of the participants (Lubkowska and Szygula, 2010).

Each treatment started with a 30 s stay in the vestibule at a temperature of -60°C. Subsequently, the subjects went directly into the main chamber, where they stayed for 3 min at -130°C. During the procedure, the participants walked slowly in a circle, one after the other (without talking, without touching), changing the walking direction every minute. Thanks to the audiovisual system, the physician supervising the course of each treatment had visual and voice contact with the individuals inside. It was possible to abort the procedure at any time.

Before going into the cryo-chamber, the participants thoroughly dried off in order to eliminate any sweat, they removed their watches, jewelry, and contact lenses. During the treatment, they were dressed in shorts, wool socks covering the ankle joints and wool pads protecting the knee joints, gloves, a headband or a hat covering the ears, clogs, and a surgical mask with gauze covering the nose and mouth.

### Blood Collection and Analyses

Venous blood was collected four times from the ulnar vessels by a qualified nurse while maintaining aseptic standards (vacuum system Vacutainer BD, United States). The first blood collection was 30 min before the first WBCST treatment. Then, blood was collected 24 h after the 12 WBCST treatment, 24 h after the 24 WBCST procedure, and 7 days after the WBCST treatment was completed. After WBCST treatments, the blood was collected at the same time with an accuracy of 15 min. Each time, the blood was collected in a seated position after 5 min of rest.

Using fluorescence flow cytometry (Sysmex XT-2000i, Japan), the following morphological indicators were determined in the whole blood: RBC content, HGB concentration, HCT, RBC indices: mean corpuscular volume (MCV), mean corpuscular HGB (MCH), mean corpuscular HGB concentration (MCHC), RBC distribution width – standard deviation (RDW-SD), RBC distribution width – coefficient of variation (RDW-CV).

In the blood plasma, the following were marked: extracellular HGB (HGB_ecf_) concentration using the cyanomethemoglobin method (Drabkin’s reagent), EPO concentration, and IL-3 concentration by immunoenzymatic assay using the R&D Systems (United States) Quantikine ELISA kits, DEP00, and D3000, respectively. The intra- and inter-assay CV of both R&D kits was less than 10%. The minimum detectable concentration for EPO was 0.6 mIU/mL and 7.4 pg/mL for IL-3.

Haptoglobin and bilirubin concentrations were determined in the blood serum using HAPT2 and BILT2 reagents (Roche Diagnostics GmbH, Germany), respectively, with the immunoturbidimetric and colorimetric methods. The intra- and inter-assay CV of both kits was less than 4.5%. The minimum detectable concentration for haptoglobin was 10 and 0.1 mg/dL for bilirubin.

### Statistical Analysis

Data distribution was checked using the Shapiro–Wilk test. The significance of inter-group differences for single measurements, depending on the distribution of variables, was tested with the Student’s *t*-test for independent trials or the Mann–Whitney *U* test. The analysis of covariance (ANCOVA) with repeated measurements was performed to compare the post-effort changes in the biochemical and hematological parameters following WBCST, depending on PA. The condition effect influence was assessed (PA), the time effect (stage of WBCST application) and the interaction of the condition and time (PA and WBCST). In the case of noting significant influence of primary factors (PA, WBCST or PA and WBCST), the significance of the differences between specific means using *post hoc* analysis (Tukey’s test) was examined. The *P* and *F* values were given. ANCOVA analysis allows to eliminate the potential influence of the associated variable (in this case, the age differences in the studied groups) on the analyzed variables. In order to document hemolytic changes and/or factors affecting the severity of erythropoiesis, correlations between growth erythropoietic factors, erythrocyte and hemolytic factors as well as mutual correlations between hemolytic indexes were calculated. Pearson’s correlations between variables were determined. For all variables, statistically significant differences were found at *P* < 0.05. All data are expressed as mean ± SD. Calculations were performed using Statistica 10 (StatSoft, Inc., Tulsa, OK, United States).

## Results

Prior to WBCST treatments, morphological content and lipid profile were comparable (*P* > 0.05) in both groups except for the concentration of high density lipoproteins which were significantly higher (*P* = 0.03) in the marathon group (1.83 ± 0.29 mmol/L) compared to the non-training group (1.41 ± 0.48 mmol/L).

No significant effects (*P* > 0.05) of WBCST, PA or interaction of these factors on RBC content, HGB concentration, HCT value or on the values of characterizing indicators of RBC (MCV, MCH, MCHC, and RDW) were noted. The baseline level for these indicators and the results of statistical analysis are presented in **Table 2**.

**Table 2:** The *F* and *P* statistics regarding the influence of physical activity (PA) and whole-body cryostimulation (WBCST) on the level of erythrocyte indicators (mean ± SD) in marathon runners and non-training men.

Variable	Marathon group	Non-training group	*F* (*P*)		
			PA	WBCST	PA × WBCST
RBC (10^6^/μL)	4.86 ± 0.50	5.05 ± 0.56	1.42 (0.25)	0.06 (0.98)	0.90 (0.45)
HGB (g/dL)	14.96 ± 1.55	15.27 ± 1.46	2.98 (0.10)	0.09 (0.87)	0.54 (0.66)
HCT (%)	43.49 ± 4.24	45.03 ± 3.99	2.87 (0.11)	0.13 (0.94)	0.78 (0.51)
MCV (fL)	89.60 ± 4.06	89.20 ± 3.12	0.03 (0.86)	0.16 (0.92)	0.62 (0.61)
MCH (pg)	30.30 ± 1.57	30.80 ± 1.03	0.56 (0.47)	0.17 (0.91)	0.69 (0.56)
MCHC (g/dL)	33.90 ± 0.69	34.38 ± 0.75	1.85 (0.19)	0.34 (0.80)	0.63 (0.60)
RDW-SD (fL)	41.80 ± 2.20	41.10 ± 2.56	0.56 (0.47)	0.48 (0.70)	0.49 (0.69)
RDW-CV (%)	12.90 ± 0.57	12.60 ± 0.52	0.69 (0.42)	0.43 (0.74)	0.42 (0.74)

*RBC, red blood cells; HGB, hemoglobin; HCT, hematocrit; MCV, mean corpuscular volume; MCH, mean corpuscular hemoglobin; MCHC, mean corpuscular hemoglobin concentration; RDW-SD, red blood cell distribution width – standard deviation; RDW-CV, red blood cell distribution width – coefficient of variation*.

**FIGURE 1:**
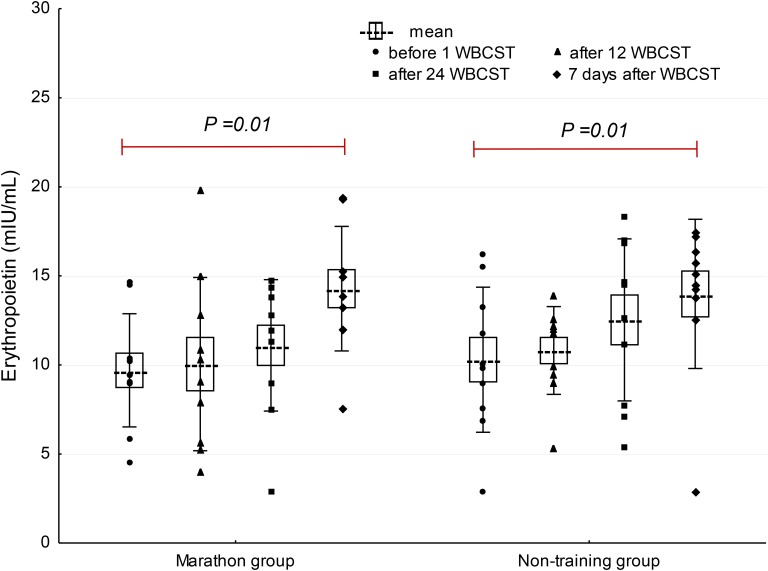
The influence of repeated whole-body cryostimulation (WBCST) treatments on the blood plasma concentration of erythropoietin in marathon runners and non-training men. Presentation of individual data, mean (---), SE (box), and SD (whiskers).

**FIGURE 2:**
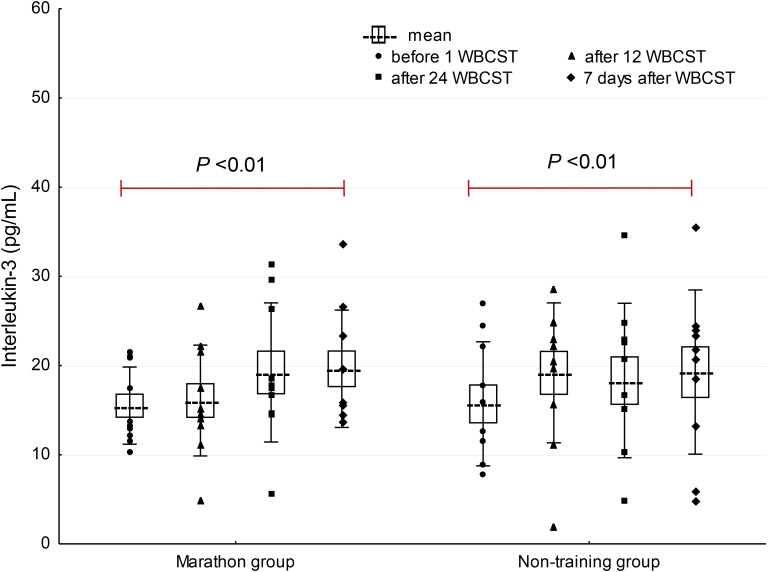
The influence of repeated whole-body cryostimulation (WBCST) treatments on the blood plasma concentration of interleukin-3 in marathon runners and non-training men. Presentation of individual data, mean (---), SE (box), and SD (whiskers).

**FIGURE 3:**
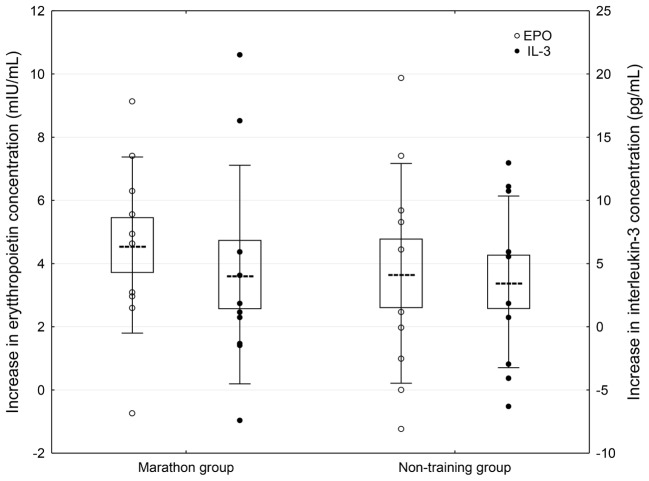
Concentration differences of erythropoietic growth factors in blood plasma 7 days after completion of 24 WBCST treatments in marathon runners and non-training men. Presentation of individual data, mean (---), SE (box), and SD (whiskers).

A significant effect of WBCST on the level of EPO (*P* = 0.01, *F* = 4.0) and IL-3 (*P* < 0.01, *F* = 4.79) were noted, while no effect of PA (*P* > 0.05), or interaction of WBCST and PA (*P* > 0.05) effects were found on these indicators. Before WBCST, the concentration of EPO was 9.70 ± 3.18 and 10.30 ± 4.07 mIU/mL, respectively in the marathon and non-training groups. The baseline level of IL-3 was 15.50 ± 4.32 pg/mL in the marathon group and 15.70 ± 6.95 pg/mL in the non-training group. The EPO and IL-3 concentrations in both groups 7 days after the end of the 24 WBCST treatments were significantly higher (*P* < 0.05) than before the first WBCST treatment. This level in the marathon and non-training groups was respectively, 14.28 ± 3.50 and 13.99 ± 4.20 mIU/mL for EPO and for IL-3: 19.63 ± 6.57 and 19.26 ± 9.21 pg/mL (**Figures 1–3**).

A significant effect of PA on changes in HGB_ecf_ (*P* = 0.01, *F* = 8.87) and haptoglobin (*P* = 0.04, *F* = 4.97) concentrations were found, but there was no WBCST effect (*P* > 0.05), or WBCST and PA (*P* > 0.05) effect. In each measurement, the HGB_ecf_ concentration was significantly higher and the haptoglobin concentration was significantly lower in the marathon group compared to the non-training group. There was no PA, WBCST effect, or any interaction of these factors (*P* > 0.05) on changes in bilirubin concentration (**Table 3**).

**Table 3:** The influence of repeated whole-body cryostimulation (WBCST) treatments on biochemical hemolytic indicators in the blood plasma of marathon runners and non-training men (mean ± SD).

Variable	WBCST	Marathon group	Non-training group	*F* (*P*)		
				PA	WBCST	PA × WBCST
HGB_ecf_ (g/dL)	Before 1 WBCST	0.70 ± 0.34	0.52 ± 0.20^†^	8.87 (0.01)	0.68 (0.57)	0.24 (0.87)
	After 12 WBCST	0.66 ± 0.22	0.49 ± 0.22^†^			
	After 24 WBCST	0.75 ± 0.28	0.51 ± 0.13^†^			
	7 Days after WBCST	0.80 ± 0.43	0.46 ± 0.16^†^			
Bilirubin (μmol/L)	Before 1 WBCST	8.09 ± 4.52	7.05 ± 3.04	1.85 (0.19)	0.65 (0.58)	0.79 (0.50)
	After 12 WBCST	9.70 ± 6.04	6.75 ± 3.49			
	After 24 WBCST	8.10 ± 4.29	6.05 ± 1.62			
	7 Days after WBCST	10.20 ± 7.42	8.88 ± 3.86			
Haptoglobin (mg/dL)	Before 1 WBCST	91.10 ± 22.82	120.70 ± 59.29^†^	4.97 (0.04)	0.47 (0.70)	0.35 (0.81)
	After 12 WBCST	86.80 ± 25.80	124.40 ± 48.45^†^			
	After 24 WBCST	89.90 ± 35.24	129.50 ± 65.67^†^			
	7 Days after WBCST	96.70 ± 43.44	120.30 ± 50.08^†^			

*Significant differences (P < 0.05). ^†^marathon runners vs. non-training men; HGB_ecf_, extracellular hemoglobin*.

There was a positive correlation (*P* < 0.05) between EPO and IL-3 concentrations (*r* = 0.35), bilirubin concentration (*r* = 0.27), MCHC (*r* = 0.25), and RDW-SD (*r* = 0.29). There was also a positive correlation (*P* < 0.05) between the concentrations of bilirubin and HGB_ecf_ (*r* = 0.25) and a negative correlation (*P* < 0.05) between haptoglobin concentration, HGB_ecf_ (*r* = -0.25) and bilirubin (*r* = -0.28).

## Discussion

Our study has shown that WBCST applied in elder men using the 12- and 24-treatment model every other day do not cause changes in RBC and HCT values or HGB concentration, nor do they induce hemolysis in marathoners or non-training individuals. Furthermore, we did not find effects of repeated WBCST on differentiation in the volume of RBCs or their hemoglobinization (mass and HGB concentration in RBC). Despite the lack of changes in the erythrocyte system, we noted that 7 days after the completion of the 24-treatment WBCST series, the concentrations of IL-3 and EPO were significantly higher compared to baseline levels. This was observed in both elder marathoners as well as men with low PA. These changes were insufficient to provoke an increase in erythropoiesis.

In the research among young men, the increase in EPO levels was achieved after 10 WBCST treatments and remained at an increased level after the series of 20 and 30 treatments, but did not affect IL-3 levels. The increase in EPO concentration was approximately 10% in the males after 30 series of WBCST (Szygula et al., 2014a). In the older males in our study, we noted a larger percentage increase in EPO concentration, i.e., approximately 47% in the marathon runners and approximately 35% in the non-training group, but only after a week after completing the WBCST series. At the same time, there was an increase in IL-3 concentration in both groups which equaled 27 and 22%, respectively. These differences may be due to the different frequency of WBCST and the age of the study participants. In the research by Szygula et al. (2014a), everyday exposure to cryogenic temperatures were used, in contrast to our model – exposure every other day.

Contrary to our findings, other researchers have reported significant reductions in RBC content, HGB concentration levels, and HCT values both in young non-training and training people (Lubkowska and Szygula, 2010; Lombardi et al., 2013; Szygula et al., 2014a; Lubkowska et al., 2015). In these studies, cryostimulation was used every day or two times a day, performing a total of 10–30 WBCST treatments. The hemolytic influence of cryogenic temperatures (-130°C) was found after single, 3-min exposure (Lubkowska et al., 2009). The damage to the erythrocyte membranes was confirmed by the increase in the concentration of free HGB in the blood (HGB_ecf_), which lasted 24 h after the end of the procedure. A significant increase in HGB levels in plasma, as well as an increase in bilirubin were also reported by Szygula et al. (2014a) after 10 and 20 WBCST treatments (daily), accompanied by a decrease in haptoglobin. A reduction in the RBC count, HGB concentration, and HCT values indicates hemolysis as a result of WBCST. The lack of hemolytic changes in older men as a result of cryostimulation in our research may be due to the used cryostimulation model. We applied repeated exposure to -130°C, but, this was done every other day. Previous studies (Szymura et al., 2016), also with the participation of older training men, showed a positive effect of this model WBCST on the rheological properties of blood, manifested by increase in erythrocyte deformability. After 12 WBCST treatments, used every other day, the elasticity of erythrocytic membranes increased, as evidenced by the higher elongation index (Szymura et al., 2016), which describes the ability of erythrocytes to shift from spherical to elliptical shape, reducing susceptibility to damage (Slowinska and Monkos, 2010).

In young subjects, an increase in the average volume of RBCs after the WBCST series was observed, with no changes in plasma volume (Lombardi et al., 2013). The increased value of red target distribution width obtained in this research indicates the presence of young, erythrocytes differentiated by size in the peripheral blood after WBCST, which indicates the activation of erythropoiesis. Similar results were obtained by Straburzynska-Lupa et al. (2007). In this study, there was an increase in MCV, MCH, and MCHC also persisting 1 week after the end of the treatments. Also, after applying a series of 20 WBCST treatments over consecutive days, men over the age of 40 who were overweight and obese, activated erythropoiesis occurred (Lubkowska et al., 2015). Under the influence of cryostimulation, there was a simultaneous reduction in mature erythrocytes and in HGB and HCT values (Straburzynska-Lupa et al., 2007; Lombardi et al., 2013; Lubkowska et al., 2015). The values of RBC, HGB, and HCT returned to baseline level only 1 month after the end of exposure to cryogenic temperatures (Lubkowska et al., 2015). These results indicate hemolysis during WBCST and consequent activation of erythropoiesis. These studies, however, do not present the results of changes in the concentration of erythropoietic growth factors.

Changes in IL-3 and EPO concentration in our research did not cause physiological effects or activation of erythropoiesis. Contrary to other researchers, we did not find other significant changes in erythrocyte indicators such as MCV, MCH, MCHC (Straburzynska-Lupa et al., 2007; Lombardi et al., 2013; Lubkowska et al., 2015), or features of erythrocyte anisocytosis (Lombardi et al., 2013; Szygula et al., 2014a). When comparing our results to those obtained by other researchers, it can be concluded that the activation of erythropoiesis is not the result of cryogenic temperatures *per se*, but is a protective mechanism against cell hypoxia as a result of hemolysis. The cryostimulation model we used did not cause hemolysis. Therefore, hemolysis is not a factor stimulating the increase in IL-3 levels in our study. The most likely cause of the delayed increase in IL-3 concentration as a result of cryostimulation in our research (1 week after the end of use) is activation of the leukocyte system, including CD4^+^ releasing IL-3 lymphocytes (Lisowska et al., 2009). In young men, the percentage of CD4^+^ T lymphocytes was increased both after a single cryostimulation and after 12 treatments (Szygula et al., 2014b). In our research, there was a significant increase in the percentage of CD4^+^ T lymphocytes, analogously to the increase in IL-3 concentration, 1 week after completion of 24 WBCST series (unpublished data). The structure of IL-3 and EPO concentration results in subsequent measurements for individual subjects in our study was not the same, both an increase and decrease in the concentration of these factors were found. The distribution of individual data was varied, which could have resulted in the lack of significant erythropoietic effects in the analysis for the whole group (Weissgerber et al., 2015).

In our research on elder marathoners and non-training individuals, in all the measurements, the RBC content, HGB concentrations, and HCT values were comparable in both groups and within the reference range for men. However, in the blood plasma of the marathon group representatives, we found a significantly higher free HGB concentration (HGB_ecf_) and at the same time, a lower haptoglobin concentration compared to men with low PA. This may indicate intravascular damage to erythrocytes in training men (Robinson et al., 2006). Contrary to studies conducted by Szygula et al. (2014a), we did not find any erythrocyte anisocytosis traits or changes in hemolytic marker concentrations such as HGB_ecf_, bilirubin, and haptoglobin during WBCST as well as 1 week after the end of treatment. The noted differences between the groups do not result from the applied cryostimulation. They may result from changes related to physical training of marathon runners. The degree of hemolysis in our marathon runners was small and did not cause clinical symptoms of anemia, as in previous studies (Fazal et al., 2017).

### Limitation of the Study

The result of the increase in IL-3 concentrations due to WBCST activity can be the activation of other non-erythropoietic cell lines (Hara and Miyajima, 1996). The EPO receptors also occur in neurons, microglia, and astrocytes of the central nervous system, as well as in endothelial cells, the smooth muscles of vessels and in myocytes and fibroblasts of the heart (Lewartowski, 2007). EPO has multidirectional action (Broxmeyer, 2013). EPO also plays an antioxidant role by influencing the activity of antioxidant enzymes and the level of Hsp70 (Maiese et al., 2008). The positive correlation found between EPO and bilirubin may be related to the protective and antioxidative activity of both molecules (Maiese et al., 2008; Suh et al., 2018). This, however, requires further research. In our research, all participants were subjected to cryogenic temperatures, the results of marathoners were referred to the non-training group. There were no comparative groups in which the training and non-training older men would not be subjected to cryostimulation. Such a study scheme would enable unambiguous determination of whether cryostimulation activates erythropoietic growth factors. Formulating conclusions on the basis of our findings related to the cause and consequences of increased IL-3 concentrations and EPO levels following repeated WBCST in training and non-training subjects is limited and requires further, detailed research taking a larger group of participants into account.

## Conclusion

Our study has shown that repeated WBCST treatments conducted every other day and 24 in total do not cause significant hemolytic blood changes in elder men with high or low PA. The WBCST model used in our study does not also result in erythropoiesis stimulation. The presented model of WBCST can be used in the elderly because it does not induce hemolytic anemia, nonetheless, the method does not increase the level of erythrocytes or their hemoglobinization. In the case of athletes, it is not a form of doping.

## Author Contributions

JS, MW, and ZS conceived the project and procured the project funding. JS, MW, MM, and JG contributed to the collection of the data and reagents for the study. MK analyzed the subjects’ diets. JS performed the data and statistical analysis. JS drafted the first version of the manuscript. All authors contributed in revising the manuscript and gave their final approval of the submitted version.

## Conflict of Interest Statement

The authors declare that the research was conducted in the absence of any commercial or financial relationships that could be construed as a potential conflict of interest. The reviewer JB and the handling Editor declared their shared affiliation.
